# Cushing's syndrome in children: An unchecked consequence of steroid use by quacks in rural areas of Pakistan

**DOI:** 10.7189/jogh.13.03039

**Published:** 2023-07-21

**Authors:** Syed M Aqeel Abidi

**Affiliations:** Medicine and Surgery, Aga Khan University Hospital, Karachi, Pakistan

As a clinical-year medical student and president of the “Humanity Initiative” non-governmental organisation (NGO), I had the opportunity to establish medical camps in rural areas of Pakistan during flood relief camps in 2022. During these camps, I encountered multiple children with features of Cushing’s syndrome; each had a history of visiting local doctors and receiving steroids. The widespread misuse by untrained practitioners has led to a surge of Cushing's syndrome cases in children and a public health crisis that needs urgent attention.

Cushing's Syndrome is a rare endocrine disorder caused by excess cortisol production in the body. It is characterised by a range of symptoms, including obesity, hypertension, diabetes, and osteoporosis. In children, the syndrome can cause growth retardation, delayed puberty, and cognitive impairment [1]. The disorder is typically diagnosed through a combination of clinical evaluation, laboratory tests, and imaging studies.

In rural areas of Pakistan, where access to medical care is limited, untrained practitioners, known as quacks, provide medical services to the local population [2], often prescribing steroids indiscriminately to treat a range of illnesses, from respiratory infections to skin conditions, without having medical training or knowledge of the medication's proper dosage, administration, or side effects. Consequently, many patients, including children, receive excessive doses of steroids, leading to the development of Cushing's syndrome.

During the flood relief camps in 2022, our medical NGO set up clinics in the affected areas to provide medical care to the flood victims. We noticed an alarming number of children presenting with symptoms of Cushing's syndrome, including excessive weight gain, moon facies, and stunted growth. Upon further investigation, we found that many of these children had been treated by local quacks with steroids for various illnesses, such as viral cough, pneumonia, and skin infections.

The situation is further complicated by the lack of awareness among the local population about the dangers of steroid misuse. Many parents believe that steroids are a quick fix for their children's illnesses and are unaware of the potential side effects of these medications. Moreover, due to the scarcity of medical facilities and qualified doctors in rural areas, many families have no other option but to rely on quacks for medical treatment.

**Figure:**
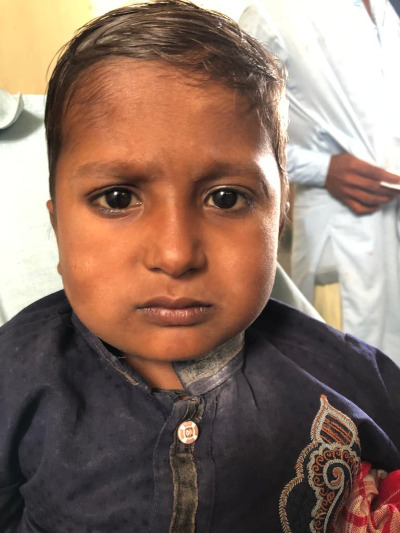
Photo: A two-year-old boy with features of Cushing's. Used with permission from child’s legal guardian.

Pakistan’s primary health care system is provided through a network of government-run health centres, clinics, and hospitals. However, these facilities often have a shortage of essential medicines, equipment, and qualified health professionals, especially in rural areas [3]. This has led to a proliferation of informal health care providers, including quacks, who often lack proper training and qualifications. It is crucial to improve the primary health care system in Pakistan to curb the practice of quackery.

The consequences of unchecked steroid use by quacks in rural areas of Pakistan are severe and long-lasting. Children developing Cushing's syndrome may suffer permanent physical and psychological damage, due to which they will require life-long medical care, and may face social stigmatisation and discrimination due to their physical appearance [4]. Moreover, caring for a chronically ill child can burden the family's finances, emotional well-being, and social status.

To address this public health crisis, there is an urgent need for a multi-pronged approach that involves raising awareness among the local population about the dangers of steroid misuse, strengthening regulatory mechanisms to prevent quackery, and improving access to qualified medical professionals in rural areas.

The unchecked use of steroids by untrained practitioners is a result of the lack of regulation of medical practices in rural areas of Pakistan. Stronger regulatory mechanisms to prevent quackery, such as stricter licensing requirements for medical practitioners, regular inspections of medical facilities, and penalties for non-compliance, will help in regulating medical practices. Regulatory strictness of pharmacies and removing steroids as over-the-counter medication can be an effective measure.

Additionally, medical NGOs and health care organisations can play a crucial role in providing medical care, educating the local population, and advocating for policy changes. Community-based health education programs that target parents, caregivers, and community leaders to raise awareness about the dangers of steroid misuse and the importance of seeking qualified medical care can be an effective and cost-effective approach.

The unchecked use of steroids by quacks in rural areas of Pakistan has led to a surge of Cushing's Syndrome cases in children, resulting in a public health crisis that needs urgent attention. Addressing this issue requires a collaborative effort from all stakeholders, including the government, health care professionals, NGOs, and local populations. By working together, we can prevent further harm to children and promote the health and well-being of the most vulnerable members of our society.
